# MiR-128 promotes osteogenic differentiation of bone marrow mesenchymal stem cells in rat by targeting DKK2

**DOI:** 10.1042/BSR20182121

**Published:** 2020-02-07

**Authors:** Can Wang, Xianghe Qiao, Zhuang Zhang, Chunjie Li

**Affiliations:** Department of Head and Neck Oncology, State Key Laboratory of Oral Diseases, West China Hospital of Stomatology, Sichuan University, Chengdu, China

**Keywords:** BMSC, DKK2, MicroRNAs, Osteogenesis, Wnt proteins

## Abstract

Bone loss caused by inflammatory disease, such as peri-implantitis, poses a great challenge to clinicians for restoration. Emerging evidence indicates that microRNAs (miRNAs) are indispensable regulators of bone growth, development, and formation. In the present study, we found that microRNA-128 (miR-128) was differentially up-regulated during the osteogenic differentiation of rat bone marrow stem cells (rBMSCs). Overexpression of miR-128 promoted osteogenic differentiation of rBMSCs by up-regulating alkaline phosphatase (ALP), matrix mineralization, mRNA, and protein levels of osteogenic makers (e.g. RUNX2, BMP-2, and COLIA1), whereas inhibition of miR-128 suppressed osteoblastic differentiation *in vitro*. Mechanistically, miR-128 directly and functionally targeted Dickkopf2 (DKK2), which is a Wnt signaling pathway antagonist, and enhanced Wnt/β-catenin signaling activity. Furthermore, the positive effect of miR-128 on osteogenic differentiation was apparently abrogated by DKK2 overexpression. Collectively, these results indicate that miR-128 promotes osteogenic differentiation of rBMSCs by targeting DKK2, which may provide a promising approach to the treatment of peri-implantitis.

## Introduction

Recently, dental implantation has been a widely accepted and common treatment option for edentulous and partially dentate patients. Successful osteointegration and long-term stability of dental implant are of significant importance for oral rehabilitation. Unfortunately, peri-implantitis, an inflammatory condition of supporting marginal bone absorption, largely threatens the maintenance of dental implants, given its high prevalence and underlying irreversible pathological changes [1,2]. Clinically, guided bone regeneration (GBR) techniques are recommended to fill bone defects and re-establish the bone-to-implant contact caused by peri-implantitis [3]. Nevertheless, the evidence is limited with regard to the success and reliability of this treatment method from a long-term survival point of view [4], which is partly due to the disrupted behavior of the local osteoblast cells in an immune-inflammatory microenvironment [5,6]. Therefore, a deep exploration of the underlying molecular mechanisms of peri-implantitis is imperative in order to optimize bone regenerative therapy.

MicroRNAs (miRNAs) are a family of non-coding RNAs that are approximately 18–22 nucleotides in length, which inhibit target gene expression by inducing mRNA degradation or by suppressing protein translation [7,8]. In addition, miRNAs are indispensable regulators that coordinate a broad spectrum of biological processes [8–10]. A series of miRNAs have been characterized as regulators of osteogenic activity and osteoblastic bone formation, either positively or negatively, through multiple signaling pathways, and the dysregulation of these miRNAs has been linked to skeletal disorders involving a reduction in bone formation. For example, miR-34a blocks osteoporosis and bone metastasis by inhibiting osteoclastogenesis and Tgif2 [11]. Moreover, miR-29a protects against glucocorticoid-induced bone loss and fragility in rats by orchestrating bone acquisition and resorption [12]. Recently, researchers have uncovered an important peri-implantitis molecular mechanism associated with miRNAs. The authors of the present study concluded that miR-27a could promote osteogenesis and angiogenesis simultaneously by targeting DKK2 and the secreted frizzled-related protein 1 (SFRP1) in a canine peri-implantitis model. The authors also successfully constructed an miR-27a-enhanced delivery system and promoted bone regeneration and osseointegration of peri-implantitis defects *in vivo*. According to miRNA sequencing results, miR-128 was differentially underexpressed in peri-implantitis compared with healthy control [13,14]. While the regulatory roles of miR-128 on various types of cancers, including lung and hepatocellular carcinoma, have been frequently reported, the study on osteogenic differentiation is scarce [15,16]. Particularly, the exact roles that miR-128 plays in the pathogenesis of peri-implantitis as well as the underlying molecular mechanisms remain unclear.

Hence, the present study was aimed at evaluating the effects of miR-128 on osteoblastic differentiation. We hypothesized that underexpressed miR-128 might be associated with the bone resorption observed during peri-implantitis. Here, we systematically explored the effects of miR-128 on rBMSCs osteogenic differentiation *in vitro* and investigated the underlying mechanism. Importantly, we identified the DKK2, a major inhibitor of the Wnt signaling pathway, as the direct target of miR-128. These results may help gain insight into the pathological mechanisms and an miRNA-based regenerative therapy for peri-implantitis.

## Materials and methods

### Cell culture

Six-week-old male Sprague-Dawley (SD) rats were obtained from the West China Hospital of Stomatology Animal Center (Sichuan, China). All the animals’ experiments were performed at Animal Center of West China Hospital of Stomatology affiliated to Sichuan University, and the ethical approval consent was obtained from the animal research committee (approval number: WCCSIRB-D-2016-019). rBMSCs were isolated and cultured as previously described [17]. Briefly, after rats were anesthetized with pentobarbital (Nembutal, 3.5 mg/100 g) and killed by cervical dislocation, both ends of rat femurs were cut off at the epiphysis, and the bone marrow was quickly rinsed out with complete medium (Dulbecco’s modified Eagle’s medium, DMEM, Gibco, Grand Island, NY, U.S.A.; 10% fetal bovine serum, FBS, HyClone Laboratories, Logan, UT, U.S.A.; 100 units/ml penicillin, and 100 µg/ml streptomycin, Gibco) followed by centrifugation at 1500 rpm for 10 min. Then, the nucleated cells were resuspended and cultured in complete medium and incubated at 37°C with 5% humidified CO_2_. After 5 days, non-adherent cells were rinsed, and fresh medium was added. The culture medium was changed every 3–4 days. When the cells reached 80–90% confluence, they were sub-cultured. Cells from passages 3–5 were used in the following experiments.

### Transfection of miRNA mimic and inhibitor

The rBMSCs were transfected with 50 nM of miR-128 mimic, 100 nM of miR-128 inhibitor, and their corresponding controls, such as mimic negative controls (mimic NCs) or inhibitor negative controls (inhibitor NCs) (Ruibo Biology; Guangdong, China) using Lipofectamine 2000 (Invitrogen; Carlsbad, CA, U.S.A.) according to the manufacturer’s instructions. Six hours after transfection, the medium was replaced. After 3 and 5 days of transfection, cells were harvested for DKK2 mRNA and protein measurement.

### Lentiviral vector construction and transduction

GFP-labeled plasmid vectors containing DKK2 and corresponding negative control (NC) were obtained from Hanbio Biotechnology (Shanghai, China). Lentiviruses were produced by transfecting 293T cells with plasmids encoding DKK2, NC, sPAX2 plasmid and pMD2G plasmid using Lipofectamine 2000. The culture medium was then changed the next day, and the supernatant was harvested after 48 h. Lentiviruses (i.e. Lenti-DKK2, Lenti-NC) were filtered and concentrated by ultrafiltration, and aliquots were stored at −80°C. For transduction, cells were incubated together with the virus (multiplicity of infection [MOI] = 20) and 5 µg/ml of polybrene (Santa Cruz Biotechnology; Santa Cruz, CA, U.S.A.) for 24 h.

### Osteogenic differentiation

To induce osteoblastic mineralization, rBMSCs were cultured in osteogenic-inducing medium (OM) containing 10 mM of β-glycerophosphate (Sigma), 100 mM of dexamethasone (Sigma), and 50 mg/ml of ascorbic acid (Sigma). The medium was then added after the transfection of mimic NC, miR-128 mimic, inhibitor NC, and miR-128 inhibitor for varying periods. For certain *in vitro* experiments, cells were also pre-treated with 10 ng/ml of IL-1β stimulation or Lenti-DKK2 and Lenti-NC.

On day 7, cells were fixed with 4% paraformaldehyde (PFA), and alkaline phosphatase (ALP) staining was performed according to the manufacturer’s instructions (Beyotime; Shanghai, China). On day 28, after fixation for 15 min, cells were incubated with 40 mM of alizarin red S (ARS) staining solution (Sigma) for 20 min at room temperature. Semiquantitative analyses of ALP activity and ARS staining were performed as previously described [18]. Briefly, after the cells were lysed, the total protein content of the samples was determined using a BCA Protein Assay Kit (Sigma). ALP activity was detected at a 405 nm wavelength with p-nitrophenyl phosphate (p-NPP) (Sigma) as the substrate. After the ARS staining was dissolved with 10% cetylpyridinium chloride (Sigma) for 1 h, the solution was distributed at 100 μl per well in a 96-well plate, and absorbance readings were taken at 590 nm using a spectrophotometer (ThermoFisher Scientific). Finally, the ALP and ARS levels were normalized to the total protein content.

### Cell growth assay

Cell proliferation rates were measured using the WST-8 Cell Counting Kit-8 (CCK-8, Dojindo; Tokyo, Japan) according to the manufacturer’s instructions. Briefly, transfected cells (2000 cells per well) were seeded in 96-well plates and incubated in DMEM supplemented with 10% FBS for 3 days. On the day of growth measurement, 100 μl of spent medium was replaced with an equal volume of fresh medium containing 10% CCK-8, and cultures were incubated at 37°C for 2 h. The absorbance was then measured at 450 nm using a microplate reader.

### Quantitative real-time PCR analysis

Osteogenic differentiation was confirmed by real-time quantitative reverse transcription PCR (qRT-PCR) examination of osteogenic-related genes, *RUNX2*, bone morphogenic protein-2 (*BMP-2*), and collagen Iα1 (*COLIA1*) in rBMSCs. The expression of miR-128 and Wnt signaling markers was also examined. Data regarding the primers used are shown in Table 1. Experiments were repeated three times per sample and the relative gene expression was calculated using the 2^−ΔΔ*C*^_t_ method, with U6 or β-actin for normalization.

**Table 1:** Primers used for qPCR

Gene name	Primer sequence (5′-3′)
DKK2	F: GGCATAGAGATCGCAACCAT
	R: GTAGGCATGGGTCTCCTTCA
Runx2	F:CTTCGTCAGCGTCCTATCAGTTC
	R:CAGCGTCAACACCATCATTCTG
BMP-2	F:GGGAGAAGGAGGAGGCAAAG
	R:GTTTCAGGGCATTTTTCAAGGT
COLIA1	F:GGAGAGTACTGGATCGACCCTAAC
	R:CTGACCTGTCTCCATGTTGCA
Lef-1	F: AATAAAGTCCCGTGGTGC
	R: ATGGGTAGGGTTGCCTGAATC
Tcf-4	F: GCCTCTCATCACGTACAGCA
	R: GGATGGGGGATTTGTCCTAC
CyclinD1	F: GTGGCCTCTAAGATGAAGGAGA
	R: GGAAGTGTTCAATGAAACGTGT
Axin2	F: TACACTCCTTATTGGGCGATCA
	R: TTGGCTAATCGTAAAGTTTTGGT
β-Actin	F: TACAGCTTCACCACCACAGC
	R: TCTCCAGGGAGGAAGAGGAT
Rno-miR-128	RT: GTCGTATCCAGTGCGTGTCGTGGAGTCG GCAATTGCACTGGATACGACAACTGT
	F: GGGTGAGGTAGTAGTTTGT
	UniRev primer: CAGTGCGTGTCGTGGAGT
U6	F: CAATACAGAGGAGATTAGCATGG
	R: GTTTCACAAATTTGCGTGTCA

### Western blot analysis

Protein lysates were generated using radioimmunoprecipitation assay lysis buffer (ThermoFisher) and nuclear protein was prepared using NE-PER Nuclear Extraction Reagents, according to the protocols specified by the manufacturer (Pierce, Rockford, IL), then protein concentrations were determined using a BCA Protein Assay Kit (ThermoFisher). Next, protein extracts (40 µg) from each sample were loaded onto a 9% sodium dodecyl sulphate-polyacrylamide gel electrophoresis (SDS-PAGE) gel and transferred to a polyvinylidene fluoride (PVDF) membrane (MilliporeSigma; Burlington, MA, U.S.A.). After blocking with 5% non-fat milk, membranes were incubated with primary antibodies against RUNX2 (1:1000), BMP-2 (1:700), COLIA1 (1:2000), DKK2 (1:1000), Lef-1 (1:500), Tcf-4 (1:500), Cyclin D1 (1:500), Axin2 (1:500), β-catenin (1:1000), and β-actin (1:2000) (all from Abcam; Cambridge, MA, U.S.A.). Immunoreactive bands were detected with secondary antibodies (1:2000) (Sigma) and visualized via enhanced chemiluminescence (Amersham Pharmacia Biotech; Little Chalfont, U.K.).

### Dual-luciferase reporter assay

A luciferase reporter assay was carried out using a Dual-Luciferase Reporter Assay System (psiCHECK-2 vector, Promega; Madison, WI, U.S.A.). A fragment of the DKK2 3′ untranslated region (UTR) containing the predicted binding site for miR-128 and the respective binding site of the mutant-type (mut) 3′ UTR were inserted into the psiCHECK-2 vector. All the constructs were verified by DNA sequencing. The psiCHECK-2 vector containing wild-type (WT) or mut was transfected into the cells with or without the synthetic miR-128 mimic. Thirty-six hours after transfection, the luciferase activity was detected using the Dual-Luciferase Reporter Assay System and normalized to Renilla activity.

### Statistical analysis

Data were expressed as the mean ± standard deviation (SD). The Student’s *t*-test or one-way analysis of variance (ANOVA) was used for multiple comparisons of the data. All experiments were repeated three times, and representative experiments were shown. All statistical analyses were carried out using the SAS 8.2 statistical software package (SAS; Cary, NC, U.S.A.). Differences were considered statistically significant at *P <0.05 and **P <0.01.

## Results

### MiR-128 is up-regulated during the osteogenic differentiation of rBMSCs

**Figure 1:**
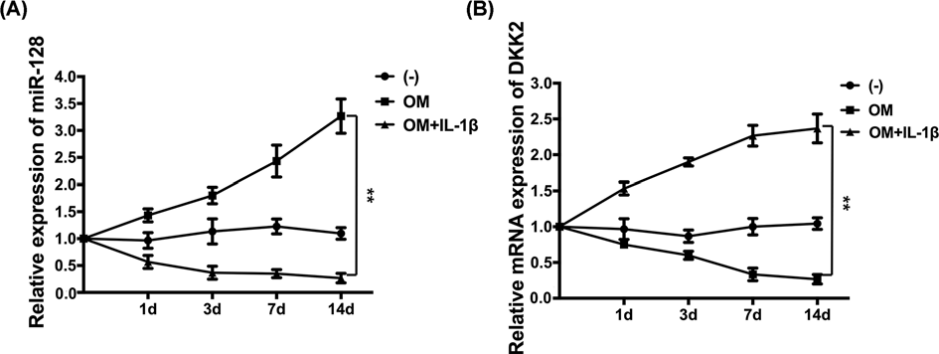
Expression levels of miR-128 and DKK2 during osteogenic differentiation of rBMSCs with or without IL-1β stimulation (**A**) Quantitation qRT-PCR analysis of miR-128 normalized to U6. (**B**) Quantitative qRT-PCR analysis of DKK2 normalized to β-actin; **P <0.01.

The inflammatory factor of IL-1β, proved to play a significant role in the onset and progression of peri-implantitis, has been used as the most important diagnostic and treatment biomarker of peri-implantitis [1]. To determine whether the down-regulation of miR-128 in peri-implantitis is related to IL-1β, we examined the expression of miR-128 under osteoinductive conditions with or without stimulation of IL-1β for 1, 3, 7, and 14 days. We found that miR-128 levels were continuously lower in the OM+ IL-1β group but continuously higher in the OM alone group compared with in the control group (Figure 1A). Furthermore, DKK2, an antagonist of Wnt signaling, was also detected during the induction process by qRT-PCR. Our results showed that when miR-128 was up-regulated during osteogenic differentiation, the expression of DKK2 was also concomitantly down-regulated, when compared with the control group (Figure 1B). Hence, we could hypothesize that the inhibitory effect of IL-1β on miR-128 expression might be one of the reasons for repressed osteoblast differentiation in peri-implantitis and that miR-128 might play a positive role during osteogenic differentiation by regulating DKK2 expression.

### MiR-128 promotes the osteogenic differentiation of rBMSCs *in vitro*

**Figure 2:**
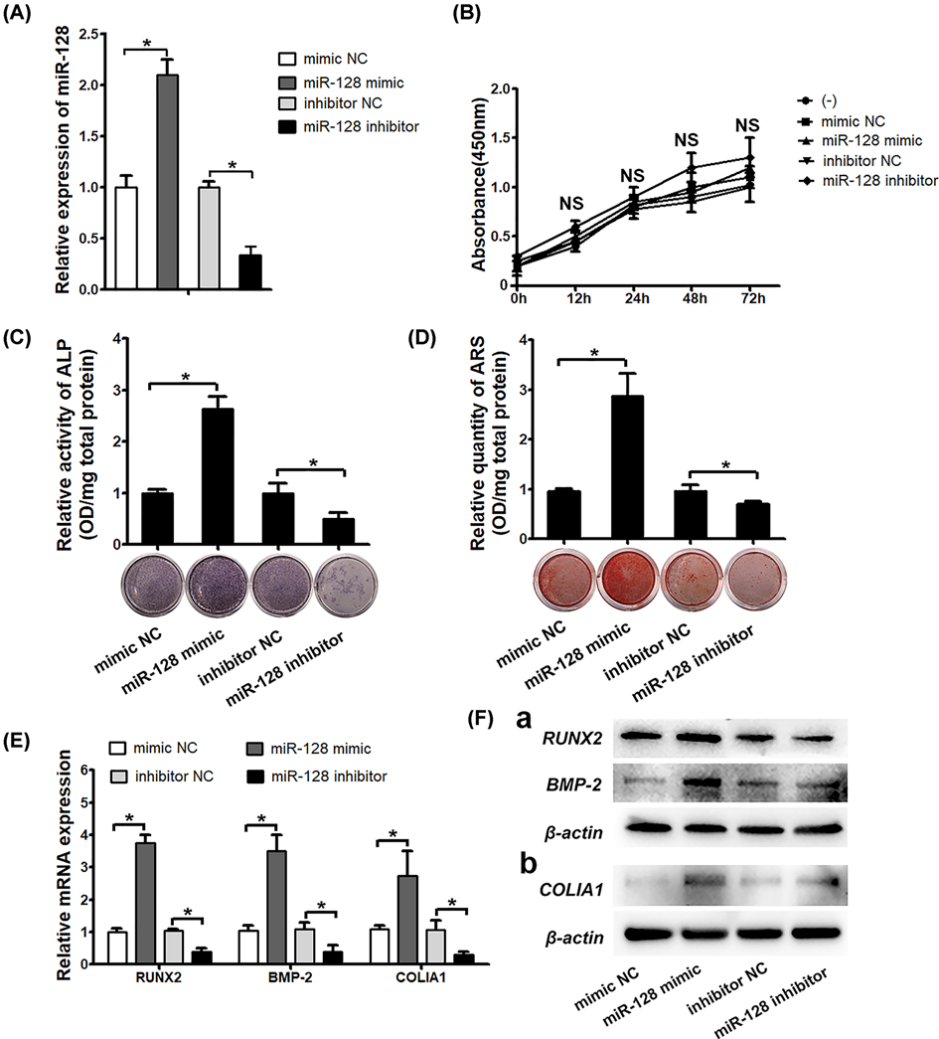
miR-128 promotes the osteogenic differentiation of rBMSCs *in vitro* (**A**) The expression levels of miR-128 examined in rBMSCs transfected with miR-128 mimic or mimic-NC, miR-128 inhibitor or inhibitor NC. (**B**) CCK-8 assay results obtained at 0, 12, 24, 48, and 72 h after miR-128 mimic or inhibitor transfection. (**C** and **D**) ALP activity and alizarin red staining performed on day 7 and 28. (**E**) Osteogenesis enhancement as evaluated by qRT-PCR analysis of osteogenic-related genes (RUNX2, BMP-2, COLIA1, normalized to β-actin) 4 and 14 days after transfection. (**F**) Western blot analysis of RUNX2, BMP-2, and COLIA1; *P < 0.05, NS, not significant.

To verify this hypothesis, rBMSCs were transfected with the mimic NC, miR-128 mimic, inhibitor NC, or miR-128 inhibitor, and a successful increase or decrease of miR-128 expression levels in these cells was verified by qRT-PCR (Figure 2A). In addition, the effect of the mimic NC, miR-128 mimic, inhibitor NC, or miR-128 inhibitor on the growth rate of rBMSCs was evaluated using a CCK-8 assay, the result of which indicated that these oligonucleotides didn’t cause obvious influence in cell proliferation (Figure 2B). After the osteogenic induction, we observed that overexpression of miR-128 strengthened the ALP activity, whereas the opposite effect was observed in miR-128–deficient cells (Figure 2C). ARS staining of the matrix mineralization revealed a similar pattern (Figure 2D).

Moreover, the expression of three pivotal osteogenic makers, RUNX2, BMP-2, and COLIA1 were detected at different time points. Our results showed that both RUNX2 and BMP-2, functioning as early-stage promoters in bone formation, were up-regulated 3.8- and 3.4-fold, respectively, by overexpression of miR-128, but reduced 2- and 2.5-fold by the knockdown of miR-128 on day 4, respectively. The other marker (COLIA1) in the late stage of osteogenesis also demonstrated the same tendency on day 14 (Figure 2E). Similarly, the protein levels of these genes were positively regulated by miR-128 (Figure 2F). Taken together, these results suggest that miR-128 is a potent candidate for the modulation of osteogenesis in rBMSCs.

### MiR-128 directly targets DKK2 and enhances Wnt/β-catenin signaling activity

**Figure 3:**
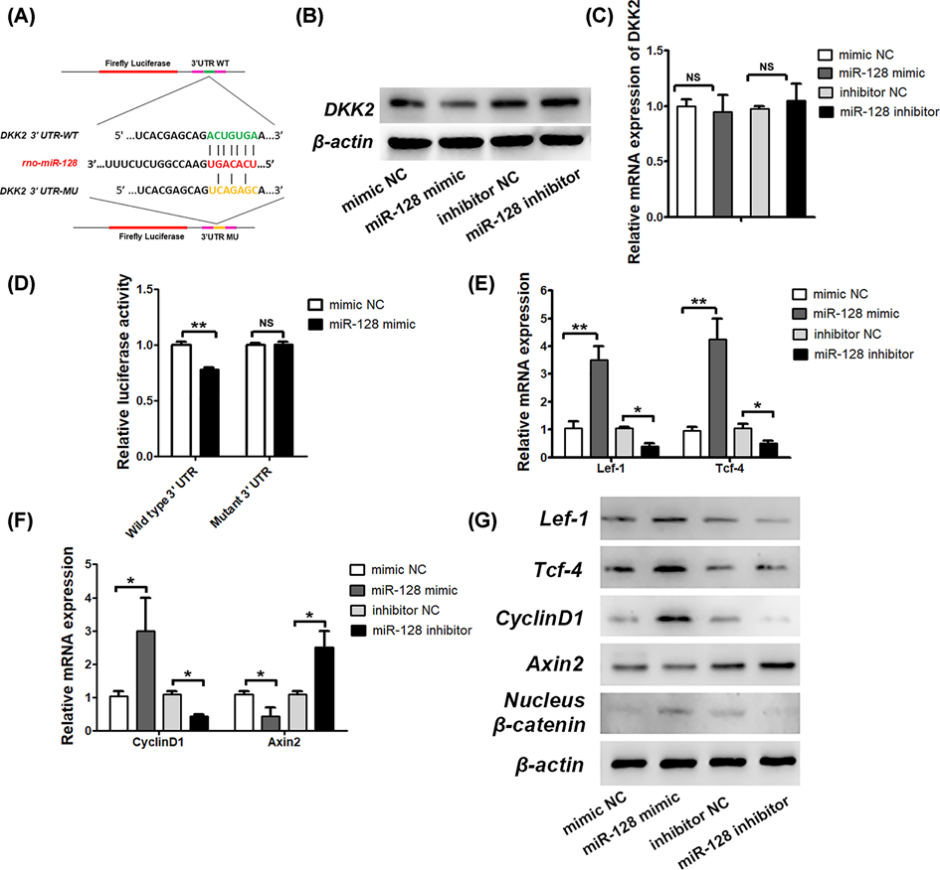
MiR-128 targets DKK2 negatively and regulates Wnt signaling pathway (**A**) Schematic diagram illustrating the design of the luciferase reporters with WT 3′-UTR or 3′-UTR-mut for DKK2. (**B** and **C**) Association analysis between miR-128 and mRNA expression levels (determined by qRT-PCR) and protein levels (determined by Western blot analysis) of DKK2. (**D**) Effects of a miR-128 mimic on luciferase activity in rBMSCs transfected with either the WT 3′ UTR reporter or the 3′ UTR-mut reporter for DKK2. (**E**) mRNA expression levels of Lef-1 and Tcf-4 as analyzed by qRT-PCR, normalized to β-actin. (**F**) mRNA expression levels of CyclinD1 and Axin2 analyzed by qRT-PCR, normalized to β-actin. (**G**) Lef-1, Tcf-4, CyclinD1, Axin2 and nucleus β-catenin protein levels were analyzed by Western blot, normalized to β-actin; **P <0.01, *P <0.05, NS, not significant.

In order to clarify the mechanisms by which miR-128 regulates osteogenic activity, we used TargetScan to predict the potential targets of miR-128. Among the candidate target genes, we found that DKK-2 has a miR-128-binding site in their 3′-UTR (Figure 3A), and levels of miR-128 and DKK2 are changed significantly in the opposite direction (Figure 1A,B). After overexpressing or inhibiting miR-128 levels in rBMSCs using the miR-128 mimic or the inhibitor, levels of the DKK2 protein decreased or increased, correspondingly (Figure 3B). However, we found no significant differences in DKK2 mRNA levels among the different groups (Figure 3C). Moreover, in order to test whether miR-128 directly targeted DKK2, we constructed luciferase reporters that had either the WT DKK2 3′-UTRs or the DKK2 3′ UTR-mut (Figure 3A). We found that the miR-128 mimic substantially inhibited the luciferase reporter activity of the WT DKK2 3′-UTR, but not that of the DKK2 with the 3′-UTR-mut (Figure 3D).

To identify a mechanism for miR-128 and Wnt signaling in rBMSCs, we monitored Wnt/β-catenin signaling activity. Here, we examined whether the transcriptional mediators of activated Wnt signaling, Lef-1 and Tcf-4, responded to miR-128. To this end, overexpression of miR-128 robustly increased endogenous Lef-1 and Tcf-4 expression, while treatment of anti-miR-128 markedly decreased Lef-1 and Tcf-4 expression (Figure 3E,G). Stimulation of Wnt signals by miR-128 through the down-regulation of Wnt pathway inhibitors is reflected by elevated Lef-1 and Tcf-4 levels. Protein levels of nucleus β-catenin were drastically enhanced by the miR-128 mimic treatment, but decreased after the miR-128 inhibitor treatment (Figure 3G), concomitantly with elevated or diminished RUNX2 as a target gene of Wnt signaling activity (Figure 2E). We also examined the expression of Cyclin D1 and Axin2, classical β-catenin target genes. Cyclin D1 was increased by the miR-128 mimic treatment and decreased after the miR-128 inhibitor treatment, while Axin2 levels decreased after the miR-128 mimic treatment and increased after the miR-128 inhibitor treatment (Figure 3F,G). Axin2 is an inhibitor and widely recognized feedback regulator of Wnt signaling. miR-128 doesn’t target Axin2 directly. miR-128 may regulate Axin2 mRNA indirectly by targeting a transcription factor that induces Axin2 [19]. These results validate that miR-128 promotes osteogenic differentiation through directly targeting DKK2 on the post-transcriptional level, thereby activating Wnt/β-catenin signaling.

### DKK2 is involved in miR-128–regulated osteoblastic differentiation of rBMSCs

**Figure 4:**
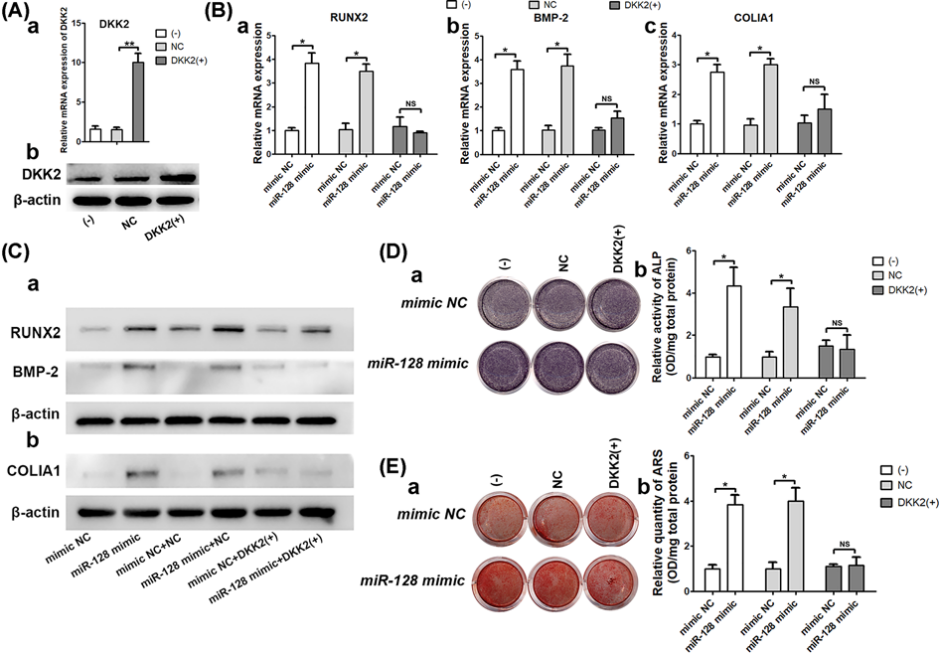
miR-128 affects osteogenic differentiation of rBMSCs by functionally targeting DKK2 (**A**) Transfection efficiencies of Lenti-DKK2 tested at 48 and 72 h after transfection using qRT-PCR and Western blot analysis, respectively. (**B**) mRNA expression of osteogenic factors as assessed after treatment with Lenti-DKK2 and miR-128 mimic. (**C**) Western blot analysis. (**D**) ALP activity. (**E**) ARS staining; **P <0.01, *P <0.05, NS, not significant.

To verify whether the effect of miR-128 during osteoblastic differentiation depended on DKK2, rBMSCs expressing Lenti-NC or Lenti-DKK2 were transfected with mimic NC or miR-128 mimic. The overexpression of DKK2 was confirmed by qRT-PCR and Western blotting (Figure 4A). After that, cells were treated with an osteoinductive medium. In our analysis, we found that the miR-128 mimic did not increase mRNA or protein expression levels of the osteogenic-related factors, RUNX2, BMP-2, and COLIA1 after DKK2 overexpression, as seen in qRT-PCR and Western blots, respectively (Figure 4B,C). Accordingly, ALP activity (Figure 4D) and matrix mineralization (Figure 4E) were also not rescued by the promotion of the miR-128 mimic when cells were transfected with the Lenti-DKK2. In summary, these findings appear to indicate that miR-128 regulates osteogenesis through functionally targeting DKK2 and activating downstream molecules of Wnt signaling pathways.

## Discussion

In peri-implantitis, activation of innate and adaptive immune responses challenged by the local bacterial infection induce the synthesis of high levels of a variety of pro- and anti-inflammatory cytokines and followed by bone resorption and formation, resulting in a net alveolar bone loss and subsequent implant failure [20]. Notably, most of the available data point to immune-inflammatory induced osteoclast differentiation and function, as the major underlying mechanism to peri-implantitis. Further, the disturbed function of bone cells and unbalanced state of bone remodeling processes may also play a role [6]. BMSCs, as the name implies, originate from bone marrow and have a great potential for regenerative therapy due to their multipotential differentiation capacity. During the process of bone formation, BMSCs are recruited from the neighboring microenvironment and differentiate into osteoblasts, regulated by complex spatiotemporal endocrine, paracrine, and autocrine interactions, involving a variety of hormones and a large number of growth factors and cytokines [21]. Importantly, miRNAs influence osteogenic differentiation, by repressing the protein translation of their targets [7]. According to a previous study, miR-128 was found to be down-regulated in a case of peri-implantitis, when compared with a healthy control group [14]. Therefore, we hypothesized that the down-regulation of miR-128 might be correlated with suppressed functions of BMSCs, thus resulting in bone loss. Hence, ascertaining the underlying molecular mechanisms mediating the regulation of BMSC osteogenic differentiation by miR-128 will help understand the pathogenesis of peri-implantitis and accelerate the development of miRNA-BMSCs-based therapies. To the best of our knowledge, we have identified for the first time, the miRNA, miR-128 as a regulator of osteoblastic differentiation of rBMSCs.

Recently, miR-128 has been widely studied in neurology, oncology, and cardiology [22–24]. With regard to regulation of MSCs differentiation, *in vitro* research has shown that miR-128 regulates the differentiation of human hair follicle mesenchymal stem cells (hHFMSCs) into smooth muscle cells (SMCs) via targeting SMAD2, a main transcription regulator in TGF-β signaling pathway involving SMC differentiation [25]. While Wu et al. has found that miR-128 acts as an endogenous attenuator of BMSCs differentiation into neurons through modulation of Wnt3a, a key component of the Wnt signaling pathway [26]. Consistent with this finding, overexpression of miR-128 in the neuronal stem cells (NSCs) prevents astrocyte differentiation and predisposes them toward neuronal differentiation [27–29]. Nonetheless, the function of miR-128 in osteogenic differentiation has not been fully investigated. In the present study, we showed that miR-128 was down-regulated during rat osteoblastic differentiation. Markedly, overexpression of miR-128 boosted osteoblastic differentiation, whereas inhibition of miR-128 expression suppressed the osteoblastic differentiation of rBMSC *in vitro*.

Precisely, how does miR-128 enhance the osteogenesis of rBMSCs? Our data strongly suggest a mechanism involving the repression of the Wnt antagonist, DKK2, although other targets are certainly possible. Notably, miR-128 overexpression down-regulated the expression of DKK2 at the protein level. Conversely, inhibition of miR-128 increased DKK2 expression. These data, together with our luciferase reporter assay results, confirm that DKK2 is a direct target of endogenous miR-128 in rBMSCs. The Wnt signaling pathway was found concordantly regulated by the overexpression and knockdown of miR-128 and presented an obvious link to rBMSCs osteogenic differentiation. In addition, our data present evidence that miR-128 directly activates Wnt signaling pathways that promote rBMSC osteogenesis. As a result of the down-regulation of DKK2 protein levels by overexpression of miR-128, Wnt signaling pathway is enhanced significantly and the transcriptional activity of β-catenin is increased. Consequently, expression levels of Wnt target genes, such as cyclinD1, Lef-1, Tcf-4, and RUNX2 are significantly up-regulated. The canonical Wnt signaling pathway is known to contribute to different stages of bone formation. Particularly, *in vivo* studies have shown that constitutive active Wnt signaling leads to increased bone mass and increased expression of bone marker genes [30–32]. Conversely, deactivation of Wnt signaling pathway by mutating LRP 5/6 produces a low bone mass phenotype and inhibits osteogenic differentiation [33,34]. Recent studies have also shown that Wnt pathways appear active during bone fracture repair and increasing the activities of Wnt pathway components can accelerate bone regeneration [35–37]. Furthermore, DKK2, as a Wnt antagonist, suppresses osteoblast activity by binding to LRP 5/6, thus preventing the formation of the Wnt-Fzd-LRP complex [38]. However, DKK2 may have a dual role in osteoblast differentiation, acting as an antagonist of Wnt canonical signaling in the early stages of osteoblast differentiation and as an agonist at later stage [5,39].

**Figure 5:**
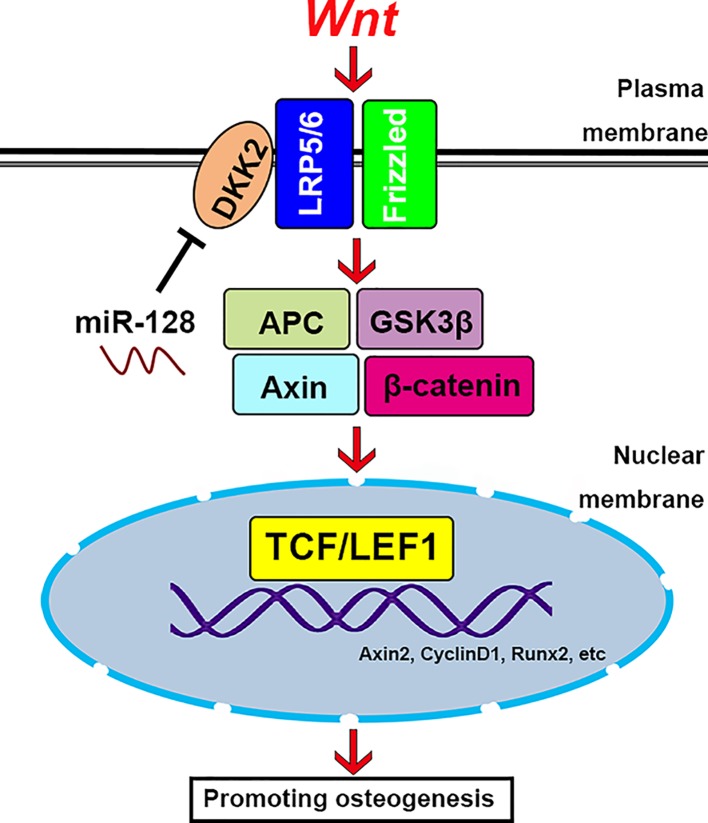
Schematic illustration depicting the mechanism by which miR-128 mediates osteogenic activity in rBMSCs

Subsequently, we tested the relationships between miR-128 and DKK2 during miR-128-promoted osteogenesis by overexpressing DKK2. The effect of miR-128-enhanced osteogenesis was not seen following the up-regulation of DKK2. Therefore, the down-regulation of DKK2 by miR-128 appear to release the brake on Wnt signaling pathway, thus activating the osteogenic capacity of Wnt signaling pathway (Figure 5).

## Conclusions

In the present study, we have characterized the roles of miR-128 in osteogenic differentiation of rBMSCs and elucidated the mechanisms of miR-128 action in this process. These findings present miR-128 as a potential target in the management of osteogenic differentiation of BMSCs for bone regeneration. In future studies, it is important to investigate whether miR-128 effectively improves the osteogenesis of BMSCs for bone regeneration *in vivo*.

## Abbreviations

ALP: alkaline phosphatase
ARS: alizarin red S
DKK2: Dickkopf2
GBR: guided bone regeneration
NC: negative control
PFA: paraformaldehyde
rBMSC: rat bone marrow stem cell
SFRP1: secreted frizzled-related protein 1
